# The Role of Cognitive and Emotional Perspective Taking in Economic Decision Making in the Ultimatum Game

**DOI:** 10.1371/journal.pone.0108462

**Published:** 2014-09-25

**Authors:** Haruto Takagishi, Michiko Koizumi, Takayuki Fujii, Joanna Schug, Shinya Kameshima, Toshio Yamagishi

**Affiliations:** 1 Tamagawa University Brain Science Institute, Tokyo, Japan; 2 Research Center for Child Mental Development, University of Fukui, Fukui, Japan; 3 Department of Brain Science, Tamagawa University, Tokyo, Japan; 4 Department of Psychology, College of William and Mary, Williamsburg, Virginia, United States of America; 5 Department of Social Welfare, Kansai University of Welfare Sciences, Osaka, Japan; 6 Graduate School of International Corporate Strategy, Hitotsubashi University, Tokyo, Japan; George Mason University / Krasnow Institute for Advanced Study, United States of America

## Abstract

We conducted a simple resource allocation game known as the ultimatum game (UG) with preschoolers to examine the role of cognitive and emotional perspective-taking ability on allocation and rejection behavior. A total of 146 preschoolers played the UG and completed a false belief task and an emotional perspective-taking test. Results showed that cognitive perspective taking ability had a significant positive effect on the proposer’s offer and a negative effect on the responder’s rejection behavior, whereas emotional perspective taking ability did not impact either the proposer’s or responder’s behavior. These results imply that the ability to anticipate the responder’s beliefs, but not their emotional state, plays an important role in the proposer’s choice of a fair allocation in an UG, and that children who have not acquired theory of mind still reject unfair offers.

## Introduction

Altruistic behavior between genetically unrelated individuals is a defining feature of human society [1]–[3]. Recently, evolutionary biologists and economists have proposed several theories to explain human altruism [4]–[6]. One theory, known as strong reciprocity, refers to the tendency to punish defectors and reward cooperators even when there is no future return for the actors [7]. The key argument of this model is that punishment toward defectors decreases incentives for selfish behavior while encouraging individuals to behave altruistically and fairly toward others. By employing economic games such as the ultimatum game (UG) [8], the dictator game, and the public goods game, a large number of experiments have shown that people tend to care about fairness, and this tendency is stronger in the presence of peer punishment [9]–[12].

The threat of peer punishment has a powerful effect on cooperative behavior. Using a public goods game, Fehr and Gächter [9] clearly demonstrated that people are sensitive to peer punishment. The public goods game is an N-person economic game in which several players simultaneously decide how much to contribute to a public fund that pays back based on the collective endowment. In their study, participants played repeated one-shot public goods games with and without the opportunity to punish other players. Cooperation rates of players gradually decreased when peer punishment was absent but increased when peer punishment was included. According to participants’ responses to a post-experimental questionnaire, almost all participants believed that low contributions to the public good would induce anger in other players. These results suggest that people anticipate that other players will become angered by and punish others who do not cooperate. Other studies have also demonstrated the effect of the threat of peer punishment in resource-allocation behavior [10]–[12].

As illustrated above, people anticipate others’ behaviors to avoid receiving punishment. Theoretically, the ability to anticipate how others will respond to one’s own behavior, as well as the ability to understand the emotions others might experience, play important roles in maintaining complex human societies. Support for this claim was first provided by Sally and Hill [13], who conducted an UG with children with and without autism spectrum disorder (ASD). The UG is a simple economic game in which two players are randomly assigned to the role of a proposer or a responder. First, the proposer receives an endowment from the experimenter and decides how to divide it between the two players. Next, the responder decides whether to accept or reject the proposer’s offer; if they accept the offer, both players receive the portion of the endowment decided by the proposer. If the responder rejects the proposer’s decision, both players receive nothing. If the proposer is motivated to maximize his or her own benefit, he or she should anticipate how the responder would respond and divide the endowment in a manner not likely to be rejected by the responder. The results of Sally and Hill’s study showed that ASD had a negative effect on the proposer’s offer: children with ASD tended to allocate less to the responder than those without ASD did.

It is well known that one of the characteristics of ASD is the lack of ability to infer others’ mental states (e.g., desires, intentions, and beliefs) [14]. This ability is known as theory of mind (ToM) [15] or mentalizing [16]. Generally, in the case of typically developing children, this ability develops between the ages of four and five years [17]. Takagishi et al. [18] recently examined the role of ToM on proposers’ behavior in the UG. In their study, three- and six-year-old preschoolers played the UG and completed a false belief task to test their acquisition of ToM. Similar to the results of Sally and Hill’s study [13], they found that ToM had a significant positive effect on proposers’ behavior: Children who had acquired ToM proposed giving more candies to the responders than children who had not acquired ToM. Furthermore, the effect of ToM remained even when the effect of age was controlled for. Preschoolers who had acquired ToM anticipated that the responder would reject unfair offers and made their allocation accordingly.

Together, these studies provide strong support for the idea that ToM, or the cognitive ability to infer the mental states of others, plays an important role in proposers’ behavior in the UG. However, a critical question remains: Is the ability to infer responders’ emotional state (i.e., emotional perspective taking) sufficient to induce proposers to make a fair offer? Recent neuroimaging and neuroendocrinological studies have shown that negative emotions drive the rejection of unfair offers in the UG [19]–[21], suggesting that responders experience negative emotion in response to unfair offers from proposers. In Takagishi et al.’s study [18], participants completed a false belief task (Sally-Anne task), which only allowed for the measuring of “cognitive” perspective-taking ability; thus, it is still uncertain whether proposers actually anticipated responders’ emotional states. Furthermore, recent studies have shown that different neural substrates underlie cognitive and emotional perspective taking [22]–[24] and that they develop separately [25]. To date, no studies have examined the role of emotional perspective-taking ability on proposers’ behavior in the UG or have compared the impact of emotional perspective-taking ability with that of cognitive perspective-taking ability. We attempted to do just this in the current study by examining the roles that cognitive perspective-taking ability and emotional perspective-taking ability play in determining proposers’ behavior in the UG. To examine the role of these abilities, we used performance on a false belief task (Sally-Anne test) and the affective perspective-taking task developed by Denham [26]. The latter has two components: an affective labeling test (ALT) and an affective perspective-taking test (APT). The ALT measures the ability to understand others’ emotional states through facial expressions, and the APT measures emotional perspective-taking ability in a social setting. We focused our analysis on the latter task and examined the relationship between the APT scores and behavior in the UG because our primary interest was emotional perspective taking (inference of another person’s emotion in a social context) rather than judging another person’s emotional state from their facial expressions.

It has been well established that adult responders’ rejection of unfair offers is based on their inference of proposers’ intentions: Very few adult responders reject an unfair offer in the UG when they are aware that the unfair offer was made unintentionally by a proposer whose choice was limited to only unfair offers [27]–[29]. This finding suggests that inference of intentionality is a prerequisite for rejecting unfair offers in the UG, and thus, cognitive perspective-taking ability, but not emotional perspective-taking ability, should be positively related to rejection behavior.

## Materials and Methods

### Ethics Statement

Parents of participants gave written consent for their preschooler’s participation in advance and the committee of Center for Experimental Research in Social Sciences, Hokkaido University approved this study.

### Participants

A total of 146 preschoolers (78 girls and 68 boys; mean age in months = 56.0, *SD* = 10.0) participated in the study. Participants were from three preschool grades: first grade (24 girls and 22 boys; mean age in months = 44.2, *SD* = 3.2; age range = 38 to 50 months), second grade (28 girls and 24 boys; mean age in months = 55.9, *SD* = 3.3; age range = 51 to 61 months), and third grade (26 girls and 22 boys; mean age in months = 67.5, *SD* = 3.8; age range = 61 to 73 months).

### Ultimatum Game

**Figure 1:**
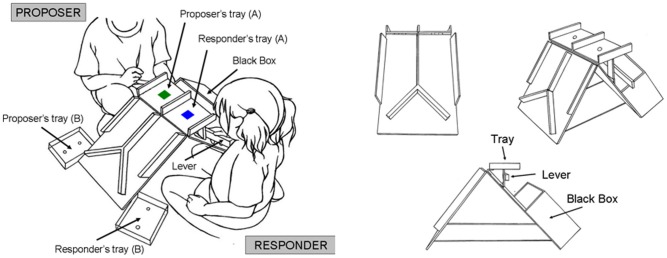
Images of the experimental apparatus. The proposer sits on the far side and the responder sits on the near side. First, the proposer makes an offer by dividing stickers between the two (proposer’s and responder’s) trays. Second, the responder decides whether to accept or reject the offer. If the responder lifts the tray, then both players receive the stickers delegated by the proposer. However, if the responder pushes the lever supporting the tray, both players receive nothing.

All participants played the UG and completed the Sally-Anne test [14] as well as Denham’s test [26]. First, seventy-three pairs matched by sex and grade played a one-shot UG. An experimenter and two preschoolers were present in the classroom during the game. In order to make the task simple enough for preschoolers, we used an experimental apparatus to aid in their understanding of the UG, and the game was conducted in a face-to-face setting without anonymity. Half of the participants were randomly assigned to the role of the proposer and half to the role of the responder. Non-monetary incentives (stickers) were used. Stickers have been widely used and their incentive value has been demonstrated in many studies investigating allocation behavior in developmental psychology [30]–[32]. The experimental apparatus was identical to that used in a previous study [18] (Figure 1). In the beginning of the game, the experimenter gave ten stickers to the proposer, who decided how to divide the stickers between themselves and the responder by placing the stickers on a tray that was divided into two sections. The proposer placed the stickers that he or she wanted to keep on the section of the tray closer to him or herself (proposer’s tray A) and placed the remaining stickers on the section closer to the responder (responder’s tray A). After the proposer made his or her allocation decision by placing the stickers on the tray, the responder decided whether to accept or reject the proposer’s offer by lifting the tray or pushing a lever. If the responder lifted the tray, the stickers on the proposer’s section of the tray slid down a ramp to the proposer’s side (proposer’s tray B), and those on the responder’s section slid down to the responder’s side (responder’s tray B). If the responder pushed the lever that supported the tray, all the stickers placed on the tray dropped down into a black box and were confiscated by the experimenter. It was clearly instructed and demonstrated to participants that once the stickers fell into the black box, neither the proposer nor the responder would receive any of them. Before starting the game, the experimenter demonstrated the tasks of the proposer and the responder to the children, who were then given the opportunity to practice using the apparatus several times.

### False Belief Task

Following the UG, participants engaged in the Sally-Anne task in order to examine their ability to understand others’ beliefs. Before the beginning of the test, the experimenter led each participant individually to an adjacent room to administer the task on a one-to-one basis. In this commonly used task, participants view a short video clip on a computer where a child (named “Natsuki” in the Japanese version) stores a ball in a box and leaves the room. While Natsuki is out of the room, another child, Yuta, moves the ball into a bag. When Natsuki returns, the participant is asked where Natsuki will look for the ball. Participants who have acquired theory of mind should understand that Natsuki would look in the box where she originally stored her ball. On the other hand, children who have not yet acquired theory of mind should assume that Natsuki would look in the bag.

### Denham’s Test

Finally, the participants completed Denham’s test in order to examine their ability to understand others’ emotional states. In the Affective Labeling Test (ALT), participants were shown four cards by the experimenter. Pictures of facial expressions of emotion (happiness, sadness, anger, and fear) were presented on each of the four cards, one at a time. Participants were then asked to name the emotion presented on the cards orally. The face cards were laid out on the floor in front of the participant, who were then asked to point at the face they showed when they felt happy, sad, angry, or fear. One point was received for each correct answer.

In the Affective Perspective-taking Test (APT), the experimenter described eight scenarios to participants by showing an animated sequence on a laptop PC and asking what emotion another child would experience in each scenario. Participants were asked to point to one of the four cards used in the ALT. In each of the eight scenarios, the protagonist experienced one of four emotions: happiness, sadness, anger, or fear (Table 1). Participants received one point per correct answer.

**Table 1:** The eight scenarios in the affective perspective-taking test.

No	Emotion	Story
1	Happiness	A child eats sweets.
2	Happiness	A mother tells a child that she will take him or her to the zoo.
3	Sadness	A friend causes a child to fall down.
4	Sadness	A child’s bicycle has disappeared.
5	Anger	A friend has pushed over a desk, which has caused the sweets on the desk to fall off.
6	Anger	A mother forces a child to eat a type of food that they hate.
7	Fear	A child has a nightmare.
8	Fear	A child becomes lost in the forest.

### Relationship Quality

In our experiment, two players played the UG in a face-to-face setting without anonymity. As relationship quality can play an important role in pro-social behavior [32], we asked the students’ teachers to rate the relationship of each of the 73 pairs on a 7 point scale (1 = very bad relationship to 7 = very good relationship) in order to examine the effect of the quality of any pre-existing relationship between the proposer and responder.

## Results

### False Belief Task and Denham’s Test

In total, four out of 46 first-grade preschoolers (9%), 23 out of 52 second-grade preschoolers (44%), and 35 out of 48 third-grade preschoolers (73%) passed the false belief task. The success rates of the false belief task were positively correlated with age in months (*r* = .55, *p*<.0001). The mean scores on Denham’s test by grade are shown in Table 2. Similar to the false belief task, the scores of ALT and APT were positively correlated with age in months (ALT, *r* = .51, *p*<.0001; APT, *r* = .53, *p*<.0001). Next, we examined the relationship between cognitive perspective taking (Sally-Anne task) and emotional perspective taking (APT). The mean scores of the APT for participants who passed and those who failed the false belief task are shown in Table 3. An ANOVA with grade (first grade, second grade, and third grade) and performance on the false belief task (passed, failed) as factors indicated a significant main effect of grade (*F*(2, 145) = 11.26, *p*<.0001, *η* ^2^ = .12), but no main effect of performance on the false belief task (*F*(1, 145) = 0.57, *p* = .45, *η* ^2^ = .00) or interaction effect (*F*(2, 145) = 0.71, *p* = .49, *η* ^2^ = .01). These results imply that while both cognitive and emotional perspective taking develop with age, they also develop independently.

**Table 2:** Mean scores on Denham’s test by grade.

	First Grade	Second Grade	Third Grade
	M (SD)	M (SD)	M (SD)
Affective Labeling Test	5.33 (1.62)	6.35 (1.63)	7.15 (0.68)
Affective Perspective-taking Test	4.70 (1.68)	5.73 (1.40)	6.85 (1.11)

**Table 3:** Mean scores of the Affective Perspective-taking Test for participants who passed and those who failed the false belief task by grade.

	First Grade		Second Grade		Third Grade	
	*N*	M (SD)	*N*	M (SD)	*N*	M (SD)
Failed the False Belief Task	*42*	4.62 (1.74)	*29*	5.76 (1.60)	*13*	6.92 (0.86)
Passed the False Belief Task	*4*	5.50 (0.58)	*23*	5.70 (1.15)	*35*	6.83 (1.20)

### Proposers’ Offers to Responders

**Figure 2:**
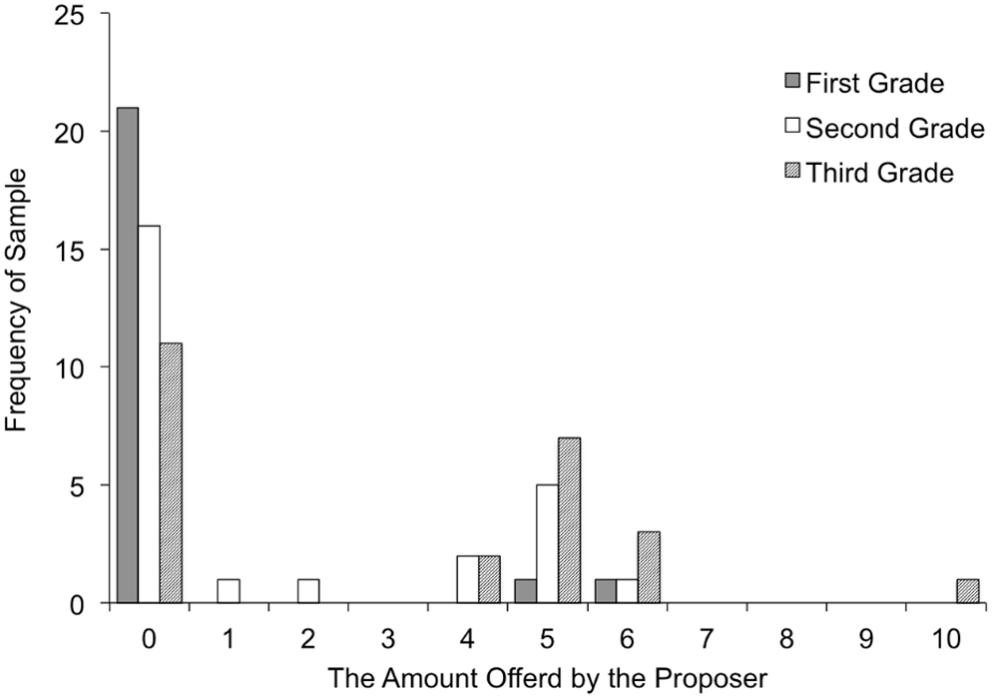
Distribution of proposers’ offers by grade.

The mean number of stickers offered to responders by grade is shown in Table 4, and the distribution of proposers’ offers by grade is shown in Figure 2. To examine the effects of cognitive and emotional perspective taking on the number of stickers offered to responders, we conducted a series of multiple regression analyses (Table 5). First, in Model 1, we regressed the number of offers to responders on age in months and sex (male = 0, female = 1) and found that age had a significant effect on the number of stickers (β = .37, p<.01), but sex did not (β = .14, p = .20). In Model 2, we added the APT, and found that it had no significant effect (β = .02, p = .91). In Model 3, we replaced APT with the false belief task (failed the false belief task = 0, passed the false belief task = 1) and found that the false belief task had a significant effect (β = .27, p<.05). In Model 4, both the false belief task and APT were included as independent variables, and we found that the effect of the false belief task remained significant (β = .27, p<.05), but the effect of the APT remained non-significant (β = .02, p = .90). The significant main effect of age in months found in Model 1 had also reduced to non-significance (β = .25, p = .11), suggesting that the increase in stickers offered to the responder was mediated by cognitive perspective-taking ability. In Model 5, we added the quality of the relationship in each of the 73 pairs as another independent variable. The results showed that the effect of the false belief task remained significant (β = .29, p<.05), but the effects of age (β = .24, p = .12), sex (β = .11, p = .32), APT (β = .01, p = .93), and the quality of relationship (β = –.06, p = .62) were not significant. Together, the results indicate that cognitive perspective-taking ability, but not emotional perspective-taking ability, played an important role in the proposer’s behavior in the UG.

**Table 4:** Mean size of offer from proposers who passed and those who failed the false belief task by grade.

	First Grade		Second Grade		Third Grade	
	*N*	M (SD)	*N*	M (SD)	*N*	M (SD)
Failed the False Belief Task	*23*	0.48 (1.59)	*15*	1.07 (1.94)	*9*	1.67 (2.50)
Passed the False Belief Task	*0*	-	*11*	2.36 (2.58)	*15*	3.73 (3.06)

**Table 5:** Multiple regression analysis using size of offer from the proposer as the dependent variable.

Independent Variables	Model 1			Model 2			Model 3			Model 4			Model 5		
	b	SE	B	b	SE	B	b	SE	B	b	SE	B	b	SE	B
Age in month	0.104	0.028	.398**	0.101	0.036	.388**	0.067	0.032	.259*	0.064	0.039	.247*	0.062	0.040	–.240*
Sex dummy(female = 0, male = 1)	0.845	0.544	.167**	0.840	0.500	.166**	0.531	0.551	.105*	0.525	0.557	.104*	0.560	0.565	–.111*
Affectiveperspective-taking test	-			0.023	0.194	.016**	-			0.025	0.190	.012*	0.018	0.191	–.013*
False belief task	-			-			1.423	0.672	.270*	1.423	0.677	.271*	1.505	0.700	–.286*
Relationshipquality	-			-			-			-			–0.148	0.300	–.055*

**p<.01,

*p<.05.

### Responders’ Decisions

**Figure 3:**
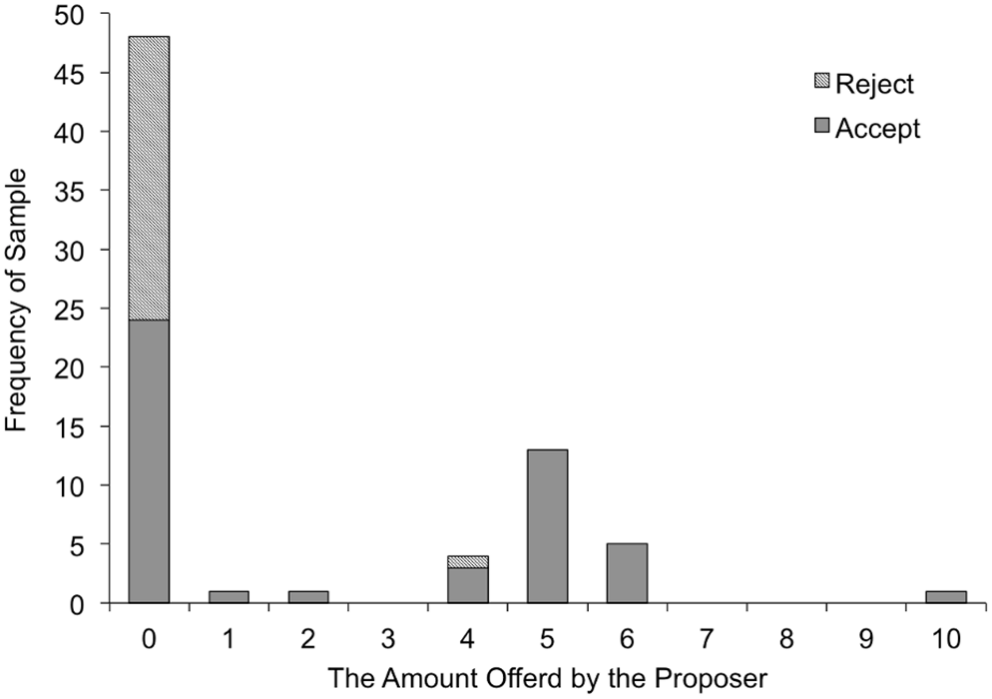
Rejection rates for each offer.

The size of the offers from the proposer was negatively correlated with rejection behavior (*r* = –.46, *p*<.0001), suggesting that unfair offers were likely to be rejected by the responders. Rejection rates for each offer are shown in Figure 3. To examine the role of cognitive and emotional perspective taking on responders’ rejection of unfair offers, we conducted a logistic regression analyses. We used the behavior of the responder as a dependent variable (acceptance of unfair offer = 0, rejection of unfair offer = 1) and age in month, sex (female = 0, male = 1), the score of APT, false belief task (failed the false belief task = 0, passed the false belief task = 1), and relationship quality as independent variables. As shown in Table 6, while emotional perspective-taking ability had no effect on rejection of unfair offers, cognitive perspective-taking ability had a weakly negative effect on rejection of unfair offers. The implications of this important finding are discussed below.

**Table 6:** Logistic regression predicting the rejection of unfair offers.

Independent Variables							
	b	SE	Wald	p	B	OR	95%CL
Age in month	–0.022	0.040	0.289	.592	–.119	1.022	0.944–1.105
Sex dummy (female = 0, male = 1)	0.709	0.649	1.192	.275	.197	0.492	0.138–1.756
Affective perspective-taking test	0.068	0.234	0.085	.771	.057	0.934	0.591–1.477
False belief task	–1.487	0.787	3.576	.059	–.413	4.426	0.947–20.68
Relationship quality	–0.112	0.372	0.090	.764	–.055	1.118	0.539–2.317

## Discussion

Our results showed that cognitive perspective-taking ability has a significant positive effect on the proposer’s offer and a negative effect on the responder’s rejection behavior, while emotional perspective-taking ability did not have any effect on either the proposer’s or the responder’s behavior. These results imply that the ability to anticipate the responder’s beliefs (such as understanding that the responder will reject unfair offers) plays an important role in the proposer’s choice of a fair allocation in the UG, while understanding of emotional states *per se* does not play a significant role in determining allocation or rejection behavior in the UG.

While surprising, the lack of an effect of emotional perspective taking observed in our study is consistent with implications of a recent brain-lesion study which found that activity in the ventromedial prefrontal cortex (vmPFC) was related to emotional perspective-taking ability but not cognitive perspective-taking ability [22]–[24]. Given this finding, patients with damaged vmPFC are expected to have low levels of emotional perspective-taking and unaffected levels of cognitive perspective-taking ability. Nevertheless, one neuroeconomic study found that the mean amount offered in an UG by patients with damaged vmPFC did not differ from those observed among patients with undamaged vmPFC [33]. These findings suggest that the lack of emotional perspective taking due to vmPFC damage does not affect the proposer’s offers in the UG. Thus, we consider our finding–that cognitive perspective-taking, but not emotional-perspective taking, is critical in making a fair offer in the UG–to be important for understanding the role of ToM, particularly cognitive perspective-taking ability, in the development of fairness-related behavior in children. However, it is premature to specify the exact reason that emotional perspective taking was not related to the proposer’s offer. One possible reason might be that emotional perspective taking is not directly related to the prediction of others’ behavior. Those who scored high on the APT understood that the responder would get angry if they made an unfair offer, yet they might not understand that the anger would cause the responder to reject their offer. This inference may require cognitive perspective-taking ability.

Another notable result of this study is that cognitive perspective-taking ability appeared to inhibit the responder’s rejection of unfair offers in the UG. On the surface, this finding contradicts the well-established finding that adult responders reject unfair offers to punish proposer’s selfish intentions [27], [28], as well as the finding that the rejection rate of unfair offers in older children is higher than rates observed in younger children [34]. As these studies suggest that cognitive perspective taking ability should enhance the rejection of unfair offers, it is puzzling that children who had cognitive perspective-taking ability did not reject unfair offers. We suspect that in the case of the current study, because the two players belonged to same class and the UG was conducted in a face-to-face setting, children who had developed cognitive perspective taking ability may have accepted unfair offers to avoid damaging their relationship with their classmate. However, as children who have not yet developed cognitive perspective taking ability cannot infer the mental states of the proposer, they would be less likely to seek to maintain relationship harmony by accepting unfair offers. Indeed, the two players in a previous study examining preschool aged children [34] did not know one another, and experiments employing the UG in adults are generally conducted in completely anonymity. Thus, in order to examine the psychological foundations of the rejection of unfair offers in children, further research is needed to compare the rejection rates between completely anonymous and in face-to-face settings.

## Supporting Information

Table S1 **Raw data.**
(XLSX)
